# Supplementary material for: A Likelihood Approach for Real-Time Calibration of Stochastic Compartmental Epidemic Models
Source: PLoS Comput Biol. 2017 Jan 17;13(1):e1005257. doi: 10.1371/journal.pcbi.1005257 (PMC5240920; doi:10.1371/journal.pcbi.1005257)
Supplement: S1 File — (TAR.GZ) [file pcbi.1005257.s014.tar.gz › HSPH_Online-SI-Revision/EnKF07/n10-severe/n10-severe_EnKF2_table4.pdf]

1	newly 3 weeks spec	197.	110.38	{3., 197.}	86.3858	0.874804	newly 3 weeks cumul	743.	735.21	{369., 830.}	67.1803	0.282351	newly 5 weeks spec	99.	81.27	{0., 181.}	121.586	1.47519	newly 5 weeks cumul	972.	911.26	{434., 1057.}	72.9424	0.327968	newly 47 weeks cumul	1325.	1267.35	{452., 2225.}	88.8264	0.426832
2	newly 3 weeks spec	183.	47.59	{0., 61.}	101.973	1.80313	newly 3 weeks cumul	725.	799.34	{33., 2057.}	129.992	0.699678	newly 5 weeks spec	116.	15.11	{0., 21.}	99.1466	2.03911	newly 5 weeks cumul	1003.	841.31	{36., 2057.}	114.639	0.764304	newly 49 weeks cumul	1262.	877.84	{36., 2057.}	106.788	0.81337
3	newly 3 weeks spec	87.	86.29	{0., 149.}	126.264	1.36581	newly 3 weeks cumul	412.	1624.89	{54., 7921.}	363.129	0.612587	newly 5 weeks spec	40.	27.24	{0., 97.}	100.75	1.28777	newly 5 weeks cumul	486.	1695.42	{67., 8380.}	316.008	0.587584	newly 51 weeks cumul	514.	1844.14	{71., 8444.}	320.677	0.576699
4	newly 3 weeks spec	158.	174.77	{53., 368.}	81.2848	0.33385	newly 3 weeks cumul	634.	1891.75	{522., 4645.}	206.879	0.363811	newly 5 weeks spec	66.	41.73	{1., 83.}	87.2273	0.856342	newly 5 weeks cumul	805.	2016.67	{573., 5217.}	163.902	0.32123	newly 46 weeks cumul	946.	2075.61	{586., 5371.}	137.525	0.293829
5	newly 3 weeks spec	156.	123.66	{0., 324.}	129.654	1.6002	newly 3 weeks cumul	639.	692.94	{30., 2207.}	112.923	0.588305	newly 5 weeks spec	112.	85.79	{0., 348.}	135.813	1.78461	newly 5 weeks cumul	896.	883.23	{46., 2809.}	114.997	0.656203	newly 46 weeks cumul	1167.	1246.85	{60., 5443.}	129.769	0.724243
6	newly 3 weeks spec	63.	254.07	{0., 383.}	404.778	1.29898	newly 3 weeks cumul	405.	1424.21	{271., 1584.}	267.262	0.479563	newly 5 weeks spec	51.	146.44	{0., 368.}	296.745	1.61776	newly 5 weeks cumul	521.	1767.05	{433., 1951.}	253.676	0.435067	newly 47 weeks cumul	643.	2221.75	{717., 4116.}	259.398	0.41876
7	newly 3 weeks spec	115.	147.41	{18., 235.}	97.5043	0.389916	newly 3 weeks cumul	383.	1276.2	{577., 2365.}	233.352	0.364988	newly 5 weeks spec	76.	37.68	{0., 72.}	80.3421	1.07879	newly 5 weeks cumul	548.	1385.66	{609., 2982.}	154.81	0.256398	newly 46 weeks cumul	668.	1433.52	{609., 2993.}	120.087	0.205234
8	newly 3 weeks spec	100.	179.97	{9., 415.}	180.19	0.554187	newly 3 weeks cumul	467.	1031.46	{116., 2521.}	153.773	0.300972	newly 5 weeks spec	34.	61.26	{0., 111.}	209.353	1.01344	newly 5 weeks cumul	561.	1198.32	{134., 2826.}	153.622	0.305106	newly 50 weeks cumul	610.	1281.58	{136., 2929.}	154.236	0.311273
9	newly 3 weeks spec	135.	115.12	{7., 89.}	128.948	0.770465	newly 3 weeks cumul	764.	351.83	{35., 317.}	100.069	0.82141	newly 5 weeks spec	57.	84.64	{2., 67.}	182.246	0.80411	newly 5 weeks cumul	905.	542.06	{43., 466.}	110.316	0.796086	newly 54 weeks cumul	1026.	793.85	{58., 923.}	118.217	0.754081
10	newly 3 weeks spec	69.	130.67	{10., 159.}	203.145	0.568179	newly 3 weeks cumul	417.	434.9	{50., 449.}	125.564	0.554448	newly 5 weeks spec	31.	92.08	{1., 103.}	309.419	0.749537	newly 5 weeks cumul	500.	642.53	{68., 745.}	148.638	0.563354	newly 54 weeks cumul	536.	864.43	{96., 1278.}	175.927	0.551879
11	newly 3 weeks spec	22.	74.43	{0., 108.}	330.5	1.00888	newly 3 weeks cumul	176.	486.29	{19., 750.}	231.097	0.515131	newly 5 weeks spec	7.	56.52	{0., 74.}	799.143	1.13986	newly 5 weeks cumul	192.	606.2	{24., 897.}	267.198	0.51841	newly 53 weeks cumul	192.	1001.9	{36., 1252.}	464.51	0.553339
12	newly 3 weeks spec	117.	52.64	{0., 95.}	88.1368	1.5029	newly 3 weeks cumul	568.	364.41	{41., 664.}	68.132	0.536011	newly 5 weeks spec	54.	43.87	{0., 89.}	109.833	1.41532	newly 5 weeks cumul	704.	457.25	{48., 1017.}	69.5412	0.537628	newly 53 weeks cumul	804.	840.25	{52., 1738.}	100.419	0.544596
13	newly 3 weeks spec	134.	197.54	{12., 392.}	143.104	0.536034	newly 3 weeks cumul	648.	1148.12	{376., 1859.}	108.843	0.203356	newly 5 weeks spec	83.	75.78	{0., 334.}	122.578	1.23287	newly 5 weeks cumul	810.	1340.37	{382., 2194.}	113.557	0.258024	newly 50 weeks cumul	934.	1585.58	{393., 3678.}	128.118	0.31983
14	newly 3 weeks spec	60.	51.94	{5., 95.}	94.9333	0.587562	newly 3 weeks cumul	328.	437.69	{102., 564.}	100.345	0.311124	newly 5 weeks spec	20.	18.73	{0., 37.}	123.05	0.989056	newly 5 weeks cumul	376.	487.35	{115., 604.}	100.178	0.331476	newly 52 weeks cumul	397.	529.37	{118., 683.}	105.469	0.348037
15	newly 3 weeks spec	54.	50.31	{0., 19.}	169.241	1.90127	newly 3 weeks cumul	371.	489.98	{159., 313.}	118.102	0.341099	newly 5 weeks spec	11.	18.99	{0., 6.}	250.818	1.82494	newly 5 weeks cumul	406.	540.83	{159., 370.}	125.5	0.367246	newly 56 weeks cumul	423.	573.88	{159., 373.}	130.993	0.382322
16	newly 3 weeks spec	69.	80.42	{1., 100.}	118.087	0.757102	newly 3 weeks cumul	368.	542.1	{41., 718.}	118.418	0.397621	newly 5 weeks spec	29.	23.18	{0., 66.}	97.2414	1.18545	newly 5 weeks cumul	440.	605.78	{41., 749.}	110.895	0.414823	newly 52 weeks cumul	501.	688.92	{41., 1010.}	112.786	0.449439
17	newly 3 weeks spec	221.	190.53	{20., 656.}	103.308	0.593053	newly 3 weeks cumul	873.	1602.64	{253., 5332.}	168.3	0.452556	newly 5 weeks spec	183.	29.17	{1., 55.}	84.0601	1.13936	newly 5 weeks cumul	1256.	1697.2	{303., 5818.}	132.947	0.486235	newly 52 weeks cumul	1842.	1745.56	{321., 5919.}	108.848	0.553658
18	newly 3 weeks spec	189.	122.11	{3., 330.}	107.095	1.00206	newly 3 weeks cumul	833.	1890.33	{100., 5456.}	179.924	0.436029	newly 5 weeks spec	133.	18.85	{0., 28.}	99.1203	2.16817	newly 5 weeks cumul	1151.	1958.35	{106., 5773.}	141.273	0.467826	newly 50 weeks cumul	1580.	1972.73	{106., 5937.}	118.863	0.543355
19	newly 3 weeks spec	134.	176.83	{0., 589.}	156.396	1.08244	newly 3 weeks cumul	645.	768.66	{44., 1827.}	107.02	0.540564	newly 5 weeks spec	78.	115.22	{0., 292.}	194.128	1.42907	newly 5 weeks cumul	830.	1027.14	{61., 2691.}	122.407	0.596936	newly 46 weeks cumul	1044.	1342.8	{81., 3225.}	137.05	0.647745
20	newly 3 weeks spec	271.	184.82	{6., 416.}	114.959	0.97885	newly 3 weeks cumul	966.	637.58	{34., 948.}	107.886	0.837163	newly 5 weeks spec	160.	125.31	{2., 357.}	127.806	1.11585	newly 5 weeks cumul	1369.	915.07	{38., 1939.}	108.61	0.877046	newly 46 weeks cumul	1954.	1528.93	{42., 7351.}	119.411	0.949522
21	newly 3 weeks spec	92.	199.39	{7., 987.}	245.685	0.752786	newly 3 weeks cumul	451.	605.95	{67., 2088.}	157.931	0.622596	newly 5 weeks spec	39.	154.08	{1., 770.}	423.436	0.928134	newly 5 weeks cumul	575.	945.38	{81., 4660.}	190.31	0.663112	newly 54 weeks cumul	717.	1353.64	{103., 7333.}	215.548	0.70127
22	newly 3 weeks spec	126.	145.09	{8., 442.}	118.865	0.632665	newly 3 weeks cumul	547.	1299.46	{164., 2354.}	206.168	0.415085	newly 5 weeks spec	54.	43.74	{0., 102.}	101.926	0.939786	newly 5 weeks cumul	674.	1409.1	{181., 2838.}	182.377	0.431405	newly 52 weeks cumul	743.	1592.73	{206., 4314.}	187.49	0.447076
23	newly 3 weeks spec	180.	187.21	{4., 372.}	129.772	0.80733	newly 3 weeks cumul	675.	995.89	{37., 1861.}	155.711	0.697426	newly 5 weeks spec	110.	70.03	{2., 116.}	106.427	0.914783	newly 5 weeks cumul	921.	1183.47	{45., 2141.}	143.995	0.720522	newly 51 weeks cumul	1186.	1319.61	{61., 2845.}	130.895	0.736988
24	newly 3 weeks spec	117.	121.32	{6., 306.}	100.12	0.566668	newly 3 weeks cumul	561.	1283.63	{192., 2096.}	195.713	0.384288	newly 5 weeks spec	36.	27.91	{0., 66.}	76.0833	0.731743	newly 5 weeks cumul	658.	1362.64	{230., 2369.}	173.556	0.375102	newly 52 weeks cumul	737.	1433.96	{245., 2436.}	160.904	0.377823
25	newly 3 weeks spec	33.	24.41	{0., 46.}	101.424	1.35408	newly 3 weeks cumul	182.	754.18	{60., 698.}	351.462	0.45217	newly 5 weeks spec	2.	12.52	{0., 29.}	625.	1.01711	newly 5 weeks cumul	198.	784.02	{71., 712.}	328.273	0.428203	newly 55 weeks cumul	198.	825.79	{78., 797.}	345.298	0.437919
26	newly 3 weeks spec	84.	193.38	{15., 389.}	213.048	0.444944	newly 3 weeks cumul	510.	636.58	{139., 957.}	121.984	0.430715	newly 5 weeks spec	55.	132.91	{4., 369.}	242.6	0.627894	newly 5 weeks cumul	628.	934.66	{178., 1929.}	144.761	0.447531	newly 54 weeks cumul	701.	1383.76	{183., 4353.}	188.348	0.464482
27	newly 3 weeks spec	183.	87.34	{0., 63.}	120.022	2.0352	newly 3 weeks cumul	804.	1066.31	{26., 3191.}	147.934	0.759238	newly 5 weeks spec	88.	38.86	{0., 24.}	119.045	1.98625	newly 5 weeks cumul	1035.	1164.36	{29., 3191.}	134.846	0.795595	newly 55 weeks cumul	1231.	1234.67	{33., 3957.}	127.206	0.823037
28	newly 3 weeks spec	70.	124.54	{8., 101.}	189.714	0.591363	newly 3 weeks cumul	412.	395.32	{44., 337.}	120.641	0.609622	newly 5 weeks spec	15.	92.89	{1., 62.}	594.067	0.600816	newly 5 weeks cumul	458.	600.72	{57., 488.}	148.166	0.578401	newly 54 weeks cumul	486.	830.04	{74., 714.}	177.844	0.542439
29	newly 3 weeks spec	27.	80.22	{6., 95.}	234.074	0.450779	newly 3 weeks cumul	254.	538.46	{240., 679.}	122.598	0.236437	newly 5 weeks spec	11.	24.6	{0., 49.}	203.818	0.719658	newly 5 weeks cumul	278.	608.68	{255., 713.}	130.388	0.241764	newly 56 weeks cumul	279.	640.26	{258., 720.}	140.695	0.252228
30	newly 3 weeks spec	46.	94.53	{3., 173.}	168.109	0.525125	newly 3 weeks cumul	317.	930.52	{139., 1106.}	221.666	0.272213	newly 5 weeks spec	3.	30.36	{0., 44.}	970.	0.949845	newly 5 weeks cumul	342.	1009.06	{141., 1259.}	224.327	0.290598	newly 56 weeks cumul	343.	1073.32	{141., 1304.}	242.099	0.306765
31	newly 3 weeks spec	146.	81.37	{0., 98.}	105.952	1.29999	newly 3 weeks cumul	569.	565.89	{36., 931.}	106.599	0.632954	newly 5 weeks spec	41.	73.54	{0., 60.}	207.366	1.30808	newly 5 weeks cumul	686.	715.37	{39., 1199.}	111.564	0.628271	newly 53 weeks cumul	786.	1246.95	{50., 5778.}	160.983	0.637983
32	newly 3 weeks spec	142.	146.59	{20., 195.}	81.7817	0.40875	newly 3 weeks cumul	533.	1391.48	{489., 2086.}	166.056	0.240379	newly 5 weeks spec	69.	39.38	{0., 86.}	71.1014	0.972862	newly 5 weeks cumul	702.	1502.15	{543., 2889.}	124.528	0.197211	newly 46 weeks cumul	971.	1579.98	{560., 3274.}	94.1442	0.199669
33	newly 3 weeks spec	184.	109.34	{5., 128.}	108.533	0.919833	newly 3 weeks cumul	700.	1310.56	{176., 2343.}	166.829	0.410069	newly 5 weeks spec	80.	11.58	{0., 25.}	92.9	1.89152	newly 5 weeks cumul	923.	1355.89	{181., 2353.}	145.349	0.480721	newly 46 weeks cumul	1232.	1364.08	{181., 2353.}	127.242	0.568604
34	newly 3 weeks spec	186.	74.59	{0., 221.}	106.758	2.37842	newly 3 weeks cumul	603.	494.32	{119., 1126.}	97.2305	0.548619	newly 5 weeks spec	102.	29.14	{0., 20.}	111.02	2.57913	newly 5 weeks cumul	831.	569.86	{119., 1230.}	96.8977	0.644579	newly 46 weeks cumul	1130.	621.28	{119., 1292.}	93.3876	0.737527
35	newly 3 weeks spec	127.	45.45	{0., 99.}	79.6929	1.42162	newly 3 weeks cumul	520.	408.67	{54., 706.}	70.0596	0.443473	newly 5 weeks spec	58.	37.54	{0., 63.}	96.8276	1.39959	newly 5 weeks cumul	660.	484.68	{74., 909.}	68.6303	0.443543	newly 53 weeks cumul	785.	817.76	{119., 1008.}	93.921	0.438532
36	newly 3 weeks spec	227.	172.08	{0., 512.}	114.907	1.68685	newly 3 weeks cumul	850.	1099.63	{63., 1665.}	117.385	0.509042	newly 5 weeks spec	131.	66.52	{0., 273.}	96.916	1.72274	newly 5 weeks cumul	1137.	1272.64	{71., 2502.}	112.628	0.567058	newly 49 weeks cumul	1502.	1440.19	{77., 4086.}	106.01	0.630202
37	newly 3 weeks spec	162.	156.2	{14., 253.}	114.247	0.620466	newly 3 weeks cumul	656.	1114.69	{179., 1930.}	157.248	0.468111	newly 5 weeks spec	79.	26.41	{0., 50.}	76.2911	0.944898	newly 5 weeks cumul	838.	1197.97	{213., 2119.}	134.342	0.466649	newly 52 weeks cumul	1008.	1274.01	{232., 2154.}	117.1	0.460142
38	newly 3 weeks spec	199.	116.6	{12., 428.}	74.804	0.583439	newly 3 weeks cumul	840.	1438.25	{245., 1932.}	157.423	0.422409	newly 5 weeks spec	85.	32.62	{1., 63.}	71.4353	0.85518	newly 5 weeks cumul	1088.	1526.17	{255., 2356.}	132.778	0.435895	newly 52 weeks cumul	1378.	1592.89	{297., 2487.}	115.422	0.461326
39	newly 3 weeks spec	207.	159.82	{39., 259.}	97.3333	0.528331	newly 3 weeks cumul	748.	613.46	{161., 949.}	70.2353	0.350828	newly 5 weeks spec	130.	92.75	{6., 155.}	110.685	0.793281	newly 5 weeks cumul	1035.	833.23	{232., 1279.}	81.0367	0.405461	newly 48 weeks cumul	1546.	1090.94	{330., 1958.}	87.4295	0.493155
40	newly 3 weeks spec	211.	180.12	{0., 801.}	108.948	1.14311	newly 3 weeks cumul	819.	1819.99	{55., 5049.}	166.287	0.464506	newly 5 weeks spec	116.	69.35	{0., 207.}	101.974	1.58649	newly 5 weeks cumul	1057.	2000.08	{64., 5400.}	146.59	0.496425	newly 48 weeks cumul	1430.	2131.25	{67., 5622.}	122.559	0.540215
41	newly 3 weeks spec	77.	109.37	{7., 403.}	142.662	0.585884	newly 3 weeks cumul	495.	920.07	{175., 2173.}	165.933	0.3698	newly 5 weeks spec	16.	36.02	{0., 54.}	213.75	0.807605	newly 5 weeks cumul	559.	1014.65	{182., 2482.}	163.182	0.385142	newly 56 weeks cumul	602.	1099.45	{182., 3035.}	166.324	0.403518
42	newly 3 weeks spec	107.	372.31	{11., 1282.}	291.523	0.535092	newly 3 weeks cumul	500.	2230.13	{132., 7070.}	380.29	0.613703	newly 5 weeks spec	44.	104.03	{1., 386.}	218.205	0.741963	newly 5 weeks cumul	615.	2525.62	{148., 8361.}	346.676	0.593445	newly 47 weeks cumul	696.	2687.14	{158., 9045.}	323.411	0.580312
43	newly 3 weeks spec	71.	46.05	{0., 48.}	119.254	1.59472	newly 3 weeks cumul	380.	707.98	{49., 1537.}	167.989	0.513778	newly 5 weeks spec	40.	21.21	{0., 24.}	116.575	1.63569	newly 5 weeks cumul	453.	759.09	{56., 1537.}	155.605	0.526179	newly 55 weeks cumul	496.	828.	{56., 2204.}	157.419	0.540499
44	newly 3 weeks spec	171.	157.25	{0., 589.}	121.596	1.66799	newly 3 weeks cumul	617.	1114.67	{165., 2432.}	121.318	0.319485	newly 5 weeks spec	118.	82.12	{0., 336.}	113.458	1.69413	newly 5 weeks cumul	869.	1310.77	{194., 3329.}	112.036	0.360354	newly 49 weeks cumul	1172.	1551.02	{222., 4748.}	113.372	0.433189
45	newly 3 weeks spec	123.	72.94	{7., 108.}	84.1138	0.651564	newly 3 weeks cumul	569.	616.85	{145., 872.}	106.533	0.427975	newly 5 weeks spec	45.	28.19	{0., 65.}	94.3778	0.908223	newly 5 weeks cumul	691.	687.27	{158., 936.}	101.009	0.44326	newly 52 weeks cumul	789.	759.96	{160., 963.}	98.8162	0.458478
46	newly 3 weeks spec	168.	292.96	{5., 1194.}	186.929	0.860362	newly 3 weeks cumul	709.	2503.58	{92., 9880.}	316.968	0.569013	newly 5 weeks spec	88.	38.3	{0., 88.}	96.0909	1.47506	newly 5 weeks cumul	909.	2648.79	{93., 10 299.}	267.196	0.600566	newly 51 weeks cumul	1016.	2670.81	{93., 10 331.}	244.094	0.613552
47	newly 3 weeks spec	173.	153.9	{10., 412.}	112.15	0.705941	newly 3 weeks cumul	651.	1104.51	{160., 2547.}	159.983	0.497479	newly 5 weeks spec	105.	30.99	{0., 58.}	79.5333	1.06972	newly 5 weeks cumul	904.	1200.31	{161., 2805.}	133.413	0.53304	newly 52 weeks cumul	1045.	1273.12	{161., 2860.}	119.841	0.52555
48	newly 3 weeks spec	241.	135.51	{31., 197.}	75.971	0.545928	newly 3 weeks cumul	934.	732.52	{162., 1912.}	76.3555	0.436376	newly 5 weeks spec	108.	61.48	{5., 86.}	87.2963	0.722001	newly 5 weeks cumul	1207.	883.48	{189., 2025.}	75.2328	0.452042	newly 49 weeks cumul	1531.	1047.56	{227., 2047.}	74.3619	0.473327
49	newly 3 weeks spec	134.	43.65	{0., 117.}	86.2761	2.04638	newly 3 weeks cumul	470.	1643.64	{47., 6965.}	298.353	0.544711	newly 5 weeks spec	57.	30.33	{0., 104.}	100.579	1.83177	newly 5 weeks cumul	619.	1710.03	{53., 6965.}	225.436	0.502098	newly 51 weeks cumul	792.	1926.33	{70., 6965.}	195.721	0.504578
50	newly 3 weeks spec	82.	110.68	{0., 103.}	168.463	1.60897	newly 3 weeks cumul	351.	1025.05	{88., 817.}	223.809	0.377408	newly 5 weeks spec	30.	57.41	{0., 41.}	224.9	1.53114	newly 5 weeks cumul	410.	1163.6	{108., 884.}	214.737	0.354898	newly 56 weeks cumul	413.	1303.76	{117., 902.}	244.722	0.366551
